# Prevalence and health outcomes of domestic violence amongst clinical populations in Arab countries: a systematic review and meta-analysis

**DOI:** 10.1186/s12889-019-6619-2

**Published:** 2019-03-18

**Authors:** Claire Hawcroft, Rachael Hughes, Amira Shaheen, Jinan Usta, Hannah Elkadi, Tom Dalton, Khadijah Ginwalla, Gene Feder

**Affiliations:** 1 Population Health Sciences, Bristol Medical School, University of Bristol, Bristol, BS8 2PS UK; 2 MRC Integrative Epidemiology Unit, University of Bristol, Bristol, UK; 3 Department of Public Health, An-Najah National University, Nablus, Palestine; 4 Department of Family Medicine, American University of Beirut Medical Center, Beirut, Lebanon

**Keywords:** Domestic violence, Intimate partner violence, Violence against women, Spouse abuse, Arab, Arabic, Middle East, Health, Healthcare

## Abstract

**Background:**

An estimated 30% of women worldwide experience intimate partner violence (IPV) during their lifetime. Exposure to IPV is associated with poor health outcomes and the prevalence of violence may be higher amongst women seeking healthcare. Existing evidence from the Arab region is limited. We conducted a systematic review and meta-analysis of prevalence and health outcomes of domestic violence (IPV or violence from a family member) in clinical populations in Arab countries.

**Methods:**

Using terms related to domestic violence, Arab countries, and date limit > year 2000, we searched seven databases: Medline, EMBASE, PsycINFO, CINAHL, Web of Science: core collection, IBSS, Westlaw, IMEMR. We included observational studies reporting estimates of prevalence or health outcomes of domestic violence amongst women aged > 15 years, recruited while accessing healthcare in Arab countries. Studies that collected data on/after 1st January 2000 and were published in English, Arabic or French were included. Title/abstract screening, full text screening, quality assessment and data extraction were carried out. Extracted data were summarised and meta-analysis was performed where appropriate.

**Results:**

6341 papers were screened and 41 papers (29 studies) met inclusion criteria. Total 19,101 participants from 10 countries were represented in the data. Meta-analysis produced pooled prevalence estimates of lifetime exposure to any type of IPV of 73·3% (95% CI 64·1–81·6), physical IPV 35·6% (95% CI 24·4–47·5), sexual IPV 22% (95% CI 13·3–32) and emotional/psychological IPV 49·8% (95% CI 37·3–62·3). Domestic violence (IPV or family violence) exposure was associated with increased odds of adverse health outcomes: depression OR 3·3 (95% CI 1·7–6·4), sleep problems OR 3·2 (95% CI 1·5–6·8), abortion OR 3·5 (95% CI 1·2–10·2), pain OR 2·6 (95% CI 1·6–4·1) and hypertension OR 1·6 (95% CI 1·2–2·0).

**Conclusions:**

Domestic violence is common amongst women seeking healthcare in Arab countries. Exposure to domestic violence is associated with several poor health outcomes. Further research into domestic violence in the Arab world is required.

**Trial registration:**

Systematic review protocol was registered on PROSPERO: CRD42017071415.

**Electronic supplementary material:**

The online version of this article (10.1186/s12889-019-6619-2) contains supplementary material, which is available to authorized users.

## Background

Domestic violence is a worldwide epidemic with an estimated 30% of women experiencing physical and/or sexual violence from an intimate partner during their lifetime [1]. Domestic violence (DV) includes violence perpetrated by a family member or intimate partner towards another adult. Much of the current international evidence focuses on intimate partner violence (IPV), which is a subset of domestic violence. Domestic violence may be a single incident or pattern of incidents which can take multiple forms including physical, sexual, psychological, emotional, financial and control violence [2]. A severe violation of human rights, domestic violence has societal and economic costs and is increasingly recognised as a clinical and public health issue.

There is a lack of robust prevalence data on domestic violence from the Arab region. A 2008 systematic review of IPV in the Middle East and North Africa highlighted ‘the very limited knowledge base on the topic’ in the region (p67) [3]. During the period 1992–2002 just 12 prevalence studies, from community or clinical settings, were identified. Estimated prevalence of lifetime exposure to IPV in these studies ranged from 8·1% to 64·6% [3]. The WHO estimated that 37% of women in the Eastern Mediterranean Region (EMR) have ever experienced physical or sexual IPV [4]. This estimate was generated from just 18 available studies compared to 114 studies used to produce the high-income countries estimate. A few countries have collected community-level prevalence data as part of large demographic health surveys; for example 30.3% of ever-married Egyptian women have experienced spousal violence, 18·6% within the last year [5]. Smaller cross-sectional studies have been conducted in community or clinical settings in some countries [6–9].

Intimate partner violence has well documented mental health, reproductive health and chronic physical health consequences [4, 10]. Two previous reviews from this region summarised the health outcomes of IPV as reported by individual clinical or community-based studies; meta-analysis was not performed [3, 11]. Evidence suggests that the prevalence of IPV may be higher amongst clinical populations, [12] but there is a lack of synthesised data internationally. We found no other studies that produced pooled prevalence estimates for *clinical* populations in the Arab region.

Domestic violence must be understood in its sociocultural context [13]. Traditional Arab society values women as wives and mothers within a patriarchal family structure, and family cohesiveness might be prioritised over individual rights and freedoms [14]. Despite progress in female education, with gender parity achieved in school-age education and women in some countries surpassing men in tertiary education, this has not necessarily translated into female economic empowerment or shifting domestic power dynamics [14–16]. Women’s subordinate status is reinforced by legal and political systems. All countries in the Arab region, except Sudan and Somalia, have now ratified CEDAW (Convention on the Elimination of all forms of Discrimination Against Women), but national responses to domestic violence have been fragmented [16, 17]. Some countries have criminalised domestic violence, but elsewhere it remains legal. In most Arab countries marital rape is not a crime. Even where penal legislation exists, survivors of domestic violence face barriers to accessing justice. Personal status law also discriminates against women with unequal access to divorce, child custody and property rights [16]. Multiple factors including family expectations, economic dependence and weak legal systems might combine to prevent women from seeking help.

With no clear consensus on which countries form the Middle East region, we chose to focus on Arab countries. These countries share sociocultural norms, making it suitable to compare and combine DV studies. Much existing international and regional prevalence data comes from community studies which provide best estimates of average prevalence. Given the health impacts of DV, women accessing healthcare may be more likely to have experienced DV. As we are particularly interested in DV as healthcare problem, it is useful for clinicians and health policy makers to have data on the prevalence and health impacts of DV within the population of women accessing healthcare. We therefore focused our review on clinical populations only. Our review aims to answer these research questions: 1. What is the prevalence of DV in clinical populations in Arab countries? 2. What are the health outcomes of DV in clinical populations in Arab countries?

## Method

### Search strategy and selection criteria

We included studies reporting prevalence or health outcomes of DV against women aged > 15 years. Population included residents of Arab countries (Bahrain, Egypt, Iraq, Jordan, Kuwait, Lebanon, Libya, Morocco, Occupied Palestinian Territory and Arab residents within Israel, Oman, Qatar, Saudi Arabia, Somalia, Sudan, Syrian Arab Republic, Tunisia, United Arab Emirates, Yemen) recruited whilst accessing healthcare. Included: observational studies, data collected on/after 1st January 2000, published in English, French or Arabic. Excluded: study population primarily migrants from non-Arab country, experimental, interventional or qualitative studies and abstracts/conference proceedings. Studies reporting health outcomes were included only if outcomes were separated or compared by violence exposure.

We included studies looking at all types of DV including physical, sexual, psychological, emotional, financial, control, *any violence* or other types. As there is no gold standard definition or measurement tool for DV prevalence, we accepted the definition/measurements used by primary studies. We included violence perpetrated by family members and/or intimate partners. Perpetration of violence within a family might occur between adult children and their parents (in either direction), from siblings, in-laws or other adult family members. Violence towards children is considered child abuse and is not included within our definition of domestic violence; we included female victims aged 15 and over, in line with the WHO (World Health Organization) approach [4]. Subsequently, we use the term DV to refer to violence from a family member and/or intimate partner and the term IPV to refer to violence from an intimate partner only.

We searched seven databases on 6^−^7th April 2017: Medline, EMBASE, PsycINFO, CINAHL, Web of Science: core collection, IBSS, Westlaw and IMEMR. Search terms used related to violence against women (VAW) and Arab countries (see Additional file 1 for full search strategy). Initial searches included all forms of VAW, study settings and EMR countries. We subsequently narrowed our focus to studies on DV, clinical populations and Arab countries only. Grey literature search included database searches (OpenGrey and ProQuest) and Google searches of government and agency reports. Backward (reference list) and forward citation (Google Scholar) searches were conducted and contact was made with authors of all included studies.

Searches, double title/abstract screening and full text review were conducted by CH and HE using EndNote. Disagreements were arbitrated by GF. French articles were also reviewed by GF.

### Data analysis

All data were extracted independently by two reviewers onto Microsoft Excel. Data extracted included study design, sampling method, response rate, sample size, inclusion/exclusion criteria, DV/IPV identification tool used, participant details, violence definition and exposure timescale. For prevalence data we recorded the number and percentage of participants exposed to different types of DV including *any violence*, physical, sexual, psychological or emotional, control, economic, or other as defined by the author. This was separated by exposure timescale: last 12 months, lifetime or during pregnancy. Health outcomes were as defined by the primary studies. Health outcome data were extracted separately for DV survivors and unexposed women.

Multiple papers from a single study were included, but only data from the paper with the largest sample or most complete data underwent analysis for each data point. For non-reported, unclear or inconsistent data, we contacted authors to seek clarification. For a small number of studies where we were unable to clarify key study design characteristics, we made the following assumptions: perpetrator of violence was IPV, timescale of abuse was lifetime, sampling method was convenience, residence was 100% urban if the clinic setting was urban. Where absolute numbers and percentages were inconsistent or could not be deduced, we used absolute numbers. There were small number of results that were judged, by two authors, to be invalid or non-comparable and were excluded from analysis. For example, a physical violence prevalence estimate which included sexual violence and child-maltreatment in the definition was not comparable to physical violence prevalence measurements from other studies. The measurement of mental health outcomes in a study which excluded patients on psychotropic medication was judged to be invalid. Where results are marked ‘N/A’ in the table in Additional file 2, this indicates that data were not considered appropriate for inclusion.

Study-level risk of bias was assessed by two authors (CH and JU) using the 20-point AXIS tool for cross-sectional studies [18]. Studies were given a global rating of poor, medium or high quality, with disagreements arbitrated by GF. One approach to variable study quality in meta-analysis is to include all studies and perform statistical adjustment based on quality assessment. As the AXIS tool does not give a numerical weighting of sources of bias, we decided that it was analytically more robust to exclude, rather than adjust for, poor-quality studies. All studies judged to be of poor-quality by two authors were therefore excluded from our review and analysis.

Prevalence studies were grouped by exposure timescale and violence type. Meta-analysis was performed if there were at least five studies in a group [19]. IPV and DV were pooled separately. For *any violence* type we only meta-analysed studies if they measured all three main violence types: physical, sexual and emotional/psychological. We fitted a random-effects model because exploratory analyses indicated substantial study heterogeneity. Prior to pooling, data were transformed using Freeman-Tukey double arcsine transformation to stabilize the variances and meet normality assumptions of the outcomes [20].

Sensitivity analysis was performed by assessing the impact of removing outlying studies on heterogeneity. We performed univariate meta-regression if there were at least five studies with relevant data: with continuous demographic covariates (age, literacy, employment and % urban population), and binary covariates (high or low/middle-income country, refugee or non-refugee population, Palestinian or non-Palestinian population, community clinic or hospital setting, women’s or general health setting, questionnaire or interview data on DV, validated or non-validated DV survey tool, short [1–9 questions] or long [10 or more questions] DV survey tool) provided there were at least two studies in each category.

We summarised health outcomes as presented by the primary studies. We generated pooled odds ratios for dichotomous health outcomes (e.g. abortion), where at least five studies provided adequate data [19]. Studies were not separated by DV or IPV, exposure timescale or violence type for the health outcome meta-analysis. Violence exposure was *any violence* type if data were available, otherwise the most prevalent violence type in that study. For univariate meta-regression, binary covariates were high or low/middle-income country and health outcome diagnostic method (validated tool or not); continuous demographic covariates used were as listed above.

Funnel plots were not used to assess for publication bias due to substantial study heterogeneity [21].

Analyses were performed using R function metaphor.

The systematic review protocol was registered on PROSPERO 21/08/2017 - CRD42017071415.

The funders had no role in study design, data collection/analysis/interpretation or report writing.

## Results

**Fig. 1:**
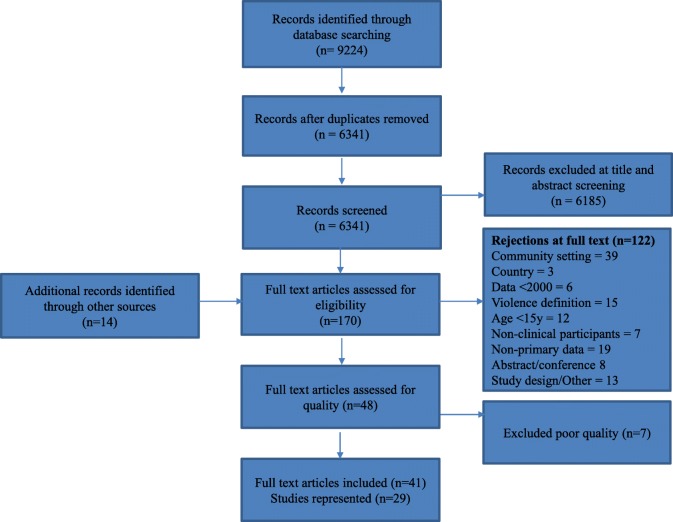
PRISMA inclusion flowchart

Searches identified 6341 papers of which 41 met inclusion criteria (Fig. 1), representing 29 studies [22–62]. Additional file 2 summarises study details and main findings from all included papers.

Data representing 19,101 participants were included in our review. Study sample size ranged from 82 to 3271, median 394. Included studies were from ten countries. Nineteen studies collected prevalence data by interview; ten used self-administered questionnaires. Seventeen studies used or modified existing validated tools for measuring domestic or intimate partner violence: 7 WHO, 3 NorAQ (NorVold Abuse Questionnaire), 2 AAS (Abuse Assessment Screen), 2 HITS (Hurt, Insult, Threaten and Scream), 1 WAST (Woman Abuse Screening Tool), 1 CTS-R (Conflict Tactics Scale, revised) and 1 USPSTF (U.S. Preventative Services Task Force) family screening tool for IPV. Twelve studies used tools which were developed independently or based on existing literature. Further detail about measurement tools is provided in Additional file 2. Most studies focused on intimate partner violence: 19 studies reported IPV only, 6 studies reported DV and 4 studies gave separate results for both IPV and DV. Further details about the perpetrators of domestic violence are included in Additional file 3. Nineteen studies reported health outcome data.

Demographic details were variably reported, with data unavailable from some studies (Table 1). Data were converted to enable comparison where possible. Participant age ranged 15–68 years. Most studies only included married/ever-married women; four included single women and one included engaged women.

**Table 1:** Demographic details as per individual studies, with average across studies

Demographic variable	Data from n = studies	Minimum	Maximum	Mean	SD
Mean age (years)	24	27·4y	36·6 y	31·4y	2·8
Currently married	23	74·3%	100%	92·8%	7·3
Literate	13	52·1%	99·6%	81·7%	13·8
Formal employment	23	0·5%	66·9%	27·2%	19
Urban residence	20	31·7%	100%	86·5%	20·7

Prevalence of DV varied between studies with wide ranging estimates for all violence types. Results are summarised by timescale of exposure below (Tables 2, 3 and 4). Descriptive summary statistics refer to IPV and DV combined. Meta-analysis results are presented separately for IPV or DV.

**Table 2:** 12-month prevalence

Violence type (12 month)	n = studies (n = pooled)	Minimum prevalence %	Maximum prevalence %	Median prevalence %	Inter-quartile range %	Pooled prevalence %	95% CI	I^2^ statistic %
**Any**	7	11·9	72	41·6	28·8–51	–	–	–
Physical	9	4	39	19·2	14·8–20·1			
	(6 IPV)					19·6	(11·7–28·8)	96·1
Sexual	4	2·1	26·4	11	3·3–20·3	–	–	–
Emotional/ psychological	8	7·5	61	37·4	15·6–50			
	(5 IPV)					38·8	(18·8–61)	99·1
Control	1	–	–	28.4	–	–	–	–
Economic	1	–	–	5.3	–	–	–	–

**Table 3:** Lifetime prevalence

Violence type (Lifetime)	n = studies (n = pooled)	Minimum prevalence %	Maximum prevalence %	Median prevalence %	Inter-quartile range %	Pooled prevalence %	95% CI	I^2^ statistic %
Any	13	30·6	89·8	61	39·3–77			
	(7 IPV)					73·3	(64·1–81·6)	97·9
Physical	17	7·7	78	31·3	26·9–44·5			
	(12 IPV)					IPV: 35·6	(24·4–47·5)	99·1
	(5 DV)					DV: 31·4	(18·4–46·1)	98·7
Sexual	11	4·3	48·3	18·8	8·8–28·8			
	(9 IPV)					22	(13·3–32)	98·7
Emotional/ psychological	14	14·7	73·4	48·8	36·7–62·8			
	(11 IPV)					49·8	(37·3–62·3)	99·1
Control	3	68·8	97·2	88·4	78·6–92·8	–	–	–
Economic	6	12	53·3	36·9	32·7–41			
	(5 IPV)					40·3	(33·0–47·8)	90·9

**Table 4:** Prevalence during pregnancy

Violence type (Pregnancy)	n = studies (n = pooled)	Minimum prevalence %	Maximum prevalence %	Median prevalence %	Inter-quartile range %	Pooled prevalence %	95% CI	I^2^ statistic %
Any	4	6·3	44·1	26·6	10·8–41·7	–	–	–
Physical	6	10·4	54	15·7	12·5–29·9	–	–	–
Sexual	4	1·2	15·5	7·9	4·6–11·4	–	–	–
Emotional/ psychological	3	23·7	32·6	28·1	25·9–30·4	–	–	–

**Fig. 2:**
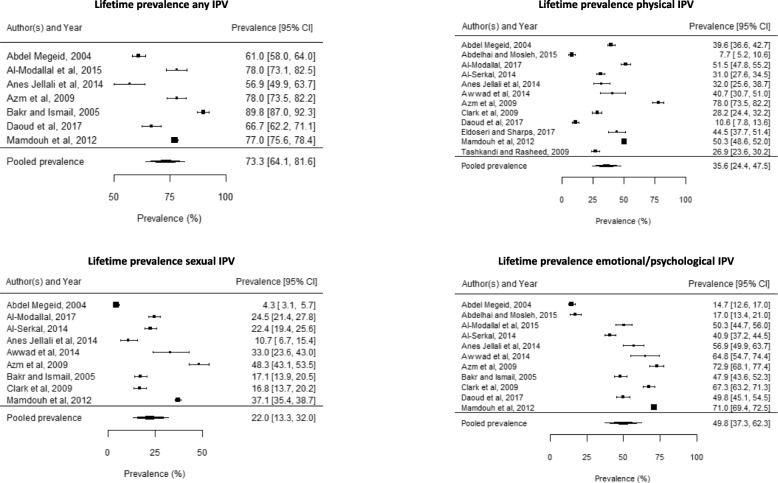
Forest plots for lifetime prevalence meta-analyses

Meta-analysis generated pooled prevalence estimates of lifetime exposure to any IPV of 73·3% (95% CI 64·1–81·6), physical IPV 35·6% (95% CI 24·4–47·5), sexual IPV 22% (95% CI 13·3–32) and emotional/psychological IPV 49·8% (95% CI 37·3–62·3). Forest plots for lifetime prevalence are presented below in Fig. 2. Forest plots for additional meta-analyses are available in Additional file 4.

For each meta-analysis, removal of outlying studies had little impact on the I^2^ statistic, so all studies were retained. Univariate meta-regression showed no consistent association between study level covariates (e.g. healthcare setting or literacy) and prevalence. Associations were detected for some analyses only: prevalence of any lifetime violence was higher in general health than women’s health settings (82% vs 66%, *p* = 0·01), prevalence of lifetime economic violence was higher in community clinics than hospitals (45% vs 33%, *p* = 0·03). Lifetime physical IPV prevalence was higher in studies using non-validated rather than validated questionnaires (52·7% vs 27·7%, *p* = 0·02) but for lifetime economic IPV, the opposite was found (36% vs 48%, *p* = 0·048). Lifetime physical DV prevalence was higher in studies with refugee populations (45%) than non-refugees (23%) (*p* = 0·041). For lifetime psychological IPV, studies with higher literacy reported higher prevalence of violence.

For health outcome data, violence exposure varied by perpetrator (IPV or DV), type of violence, and timescale. The measurement of health outcomes varied between self-reported, clinically diagnosed or measured using a screening/diagnostic tool. Additional file 5 summarises the health outcome data presented by primary studies. Most studies collected binary or categorical data and reported unadjusted odds ratios (OR); some used other statistical measures. Odds ratios were recalculated from absolute figures where available. Pooled effect estimates were produced for the five health outcomes for which there were sufficient data: depression, sleep, abortion, pain and hypertension (Table 5). Studies were combined in meta-analysis regardless of whether the study measured IPV or DV, length of exposure timescale or violence type.

**Table 5:** Health outcome meta-analysis results

Health outcome	n = studies	Pooled OR	95% CI	I^2^ statistic
Depression	5	3·3	1·7–6·4	68·1
Sleep problems	5	3·2	1·5–6·8	87·4
Abortion	6	3·5	1·2–10·2	94·1
Pain	5	2·6	1·6–4·1	74·0
Hypertension	5	1·5	1·2–2·0	27·3

**Fig. 3:**
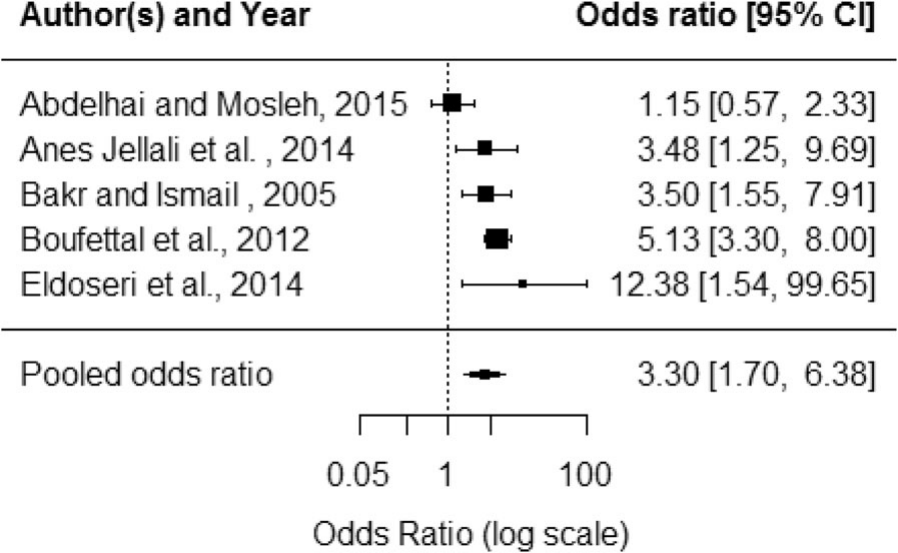
Depression forest plot

**Fig. 4:**
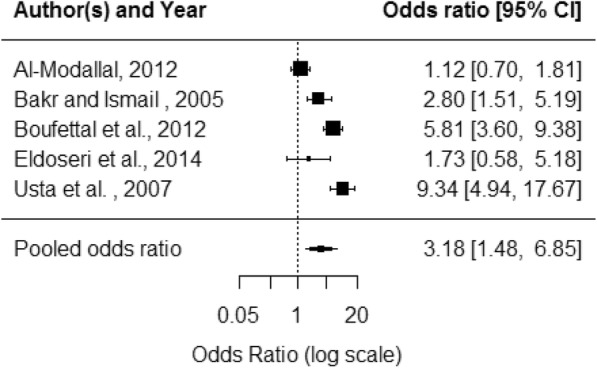
Sleep problems forest plot

Health outcomes were grouped into mental health, reproductive health and general health. Depression was the mental health outcome with the most consistent evidence: seven studies all found a significant association with DV. Meta-analysis of five of these studies produced a pooled OR of 3·3 (95% CI 1·7–6·4) (Fig. 3). Seven studies considered sleep problems with five finding significant associations. Pooled OR for five of the seven studies was 3·2 (95% CI 1·5–6·8) (Fig. 4). Other mental health outcomes found to be associated with domestic violence by some studies were suicidal thoughts, suicide attempts, anxiety, anxiety/depression, postnatal depression, stress, memory problems, distress and anguish. See Additional file 5 for more detail.

**Fig. 5:**
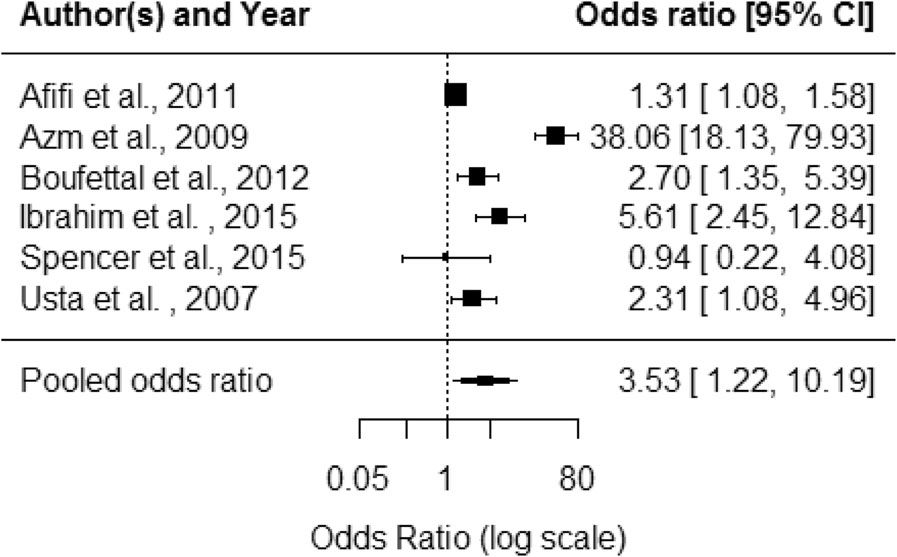
Abortion forest plot

For reproductive health, there was a mix of significant and non-significant results for most outcomes including vaginal bleeding, unplanned pregnancy, premature labour, contraceptive use, stillbirth and premature rupture of membranes. Seven studies looked at abortion with all but one finding a significant association. Not all papers defined whether abortion was spontaneous or induced. Meta-analysis for abortion produced a pooled OR of 3·5 (95% CI 1·2–10·2) (Fig. 5).

**Fig. 6:**
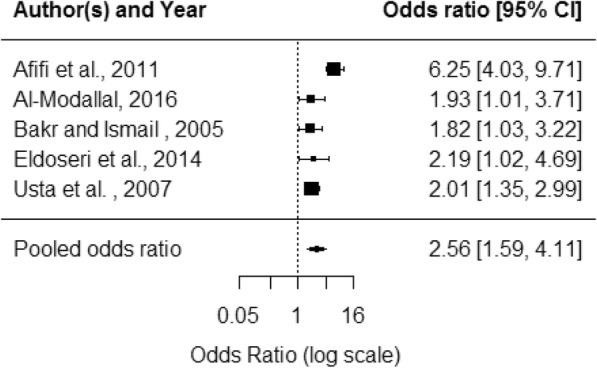
Pain forest plot

**Fig. 7:**
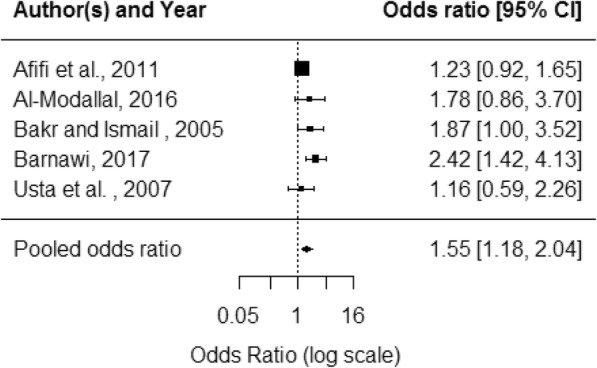
Hypertension forest plot

General health outcomes included clinical diagnoses such as diabetes, symptoms such as pain and measures of general wellbeing such as mobility. Most data were from patient-reported measures. For most outcomes, there was a mixture of significant and non-significant results. Six studies looking at pain all found significant associations; Al-Modallal found associations for some types of violence but not others [30]. Meta-analysis produced a pooled OR of 2·6 (95% CI 1·6–4·1) (Fig. 6). Five studies looked at hypertension with two studies finding significant associations (for some but not all types of violence); meta-analysis produced pooled OR of 1·6 (95% CI 1·2–2·0) (Fig. 7).

For most covariates there was insufficient data to perform univariate meta-regression. Of the analyses performed, two showed significant associations. Odds of abortion in women exposed to DV were higher in studies with higher employment levels. Odds of pain in women exposed to DV were higher in studies set in high-income (OR 4·3) than low/middle-income countries (OR 1·93).

## Discussion

This is the first systematic review and meta-analysis of the prevalence and health outcomes of domestic violence in clinical settings in the Arab region. Around one in two women experienced psychological violence, one in three physical violence and one in five sexual violence from an intimate partner during their lifetime, with over 70% experiencing any form of violence. These rates are higher than community-level estimates from local demographic and health surveys (despite broadly comparable baseline characteristics). Around one in three women in Iraq have experienced lifetime emotional IPV, one in four women in Egypt experienced lifetime physical IPV and one in ten women in Jordan experienced lifetime sexual IPV [5, 63, 64]. This may indicate a higher prevalence of DV amongst women seeking healthcare.

Internationally, there is a lack of pooled DV prevalence data from clinical settings. A UK-based systematic review found lifetime IPV prevalence reported by individual studies ranging from 13% in a maternity service to 41% in primary care [12]. One meta-analysis, including primarily USA and high-income country studies, estimated lifetime exposure to any type of IPV of 38% in family medicine and 40% in emergency medicine settings [65]. Our study is the first to produce pooled estimates of IPV prevalence in clinical populations in the Arab region, or indeed any low/middle-income setting. Understanding the prevalence of DV/IPV amongst the population of women attending healthcare settings can inform adaptations to clinical care and health service provision.

We found mixed evidence of associations between DV and a wide range of general, reproductive and mental health outcomes. This is the first study to present pooled effect estimates of the health outcomes of DV in the Arab region. Exposure to DV significantly increased the odds of the five adverse health outcomes that we meta-analysed. These figures are higher than WHO estimates of associations between physical and/or sexual IPV and incident depression OR 1·97, induced abortion OR 2·16 and pain (last 4 weeks) aOR (adjusted odds ratio) 1·6 [4, 66]. We found no existing pooled effect estimates for sleep or hypertension.

Strengths of this review include a pre-defined protocol published online, a broad search strategy and inclusion of publications in languages other than English. Two authors completed each review stage, thereby minimising bias. Quality assessment was conducted by two authors using a published checklist. We used robust statistical methods and provide justification of our analysis decisions.

The reliability of our results is limited by the quality of the primary studies. Most were small to medium sized cross-sectional studies; only a minority justified their sample size. Where this was done, and specified, studies were more often powered to detect prevalence rather than associations with health outcomes (which would require a larger sample). Sampling was by convenience in most studies which may introduce selection bias. Some studies had non-representative populations e.g. large proportion of military police husbands [42], high level of female employment [40], or only refugee participants [27, 28, 30, 34, 35, 52, 53, 55]. Twenty-nine papers reported response rates (which were generally high) but few assessed for non-response bias. The majority of studies either gained institutional ethics approval or at least gave some consideration to ethical issues. Best practice, according to WHO research recommendations, includes reducing distress to research participants and referring for additional support if required; few studies made explicit mention of this [67].

The lack of a gold standard definition of IPV or DV hinders this research field. Terms for categories of violence against women may be overlapping or used interchangeably. Studies in this review used varied methods for measuring violence. Face-to-face interviews versus self-administered questionnaires may impact prevalence and recall bias may be affected by length of recall period. International measurement tools require translation then revalidation. Pre-existing tools may not include behaviours relevant to the Arab setting e.g. ‘cursing wife’s family’. [40] Clark and colleagues used focus groups to inform modifications to the WHO questionnaire; identification of control violence increased from 83 to 97% [45]. Psychological, emotional and control violence are particularly challenging to define. Some studies included what might be considered low-level behaviours e.g. shouting, ‘taking his own decision’, ‘being threatened with divorce’ and produced high estimates of psychological or control violence [40, 41, 57]. It may be the frequency and combination of these behaviours (measured by a few of the primary studies) which determines impact. Rates for control violence ranged from 69 to 97%, comparable to 83% in the Iraq Family Health Survey [63]. Control has been increasingly recognised internationally as a specific type of domestic violence, with some countries now criminalising coercive control [68]; control was included as defined and measured by a small number of studies in our review. Sexual violence can be difficult to measure and may be underestimated; one study removed questions due to perceived cultural sensitivity [26].

There remain gaps in the evidence on DV in clinical populations in Arab countries. Only ten out of the nineteen countries searched for are represented in our review. We retrieved no relevant Palestinian studies so could not conduct the separate analysis pre-specified in our protocol. Most studies focused on IPV, rather than the broader scope of all family violence. Studies which did look at DV (violence from any adult family member) reported in varying detail on the perpetrators of abuse (see Additional file 3). Husbands remained the most frequent abusers, sometimes in combination with other family members. In some studies and for some violence types, mother-in-laws were the most common (non-husband) family perpetrators [25, 47, 59], and in some studies brothers or fathers were the most common [25, 34, 35, 51]. Other perpetrators included mother, father-in-law, other in-laws and other wives. One study found differences in the type of abuse perpetrated by different family members with husbands responsible for the majority of sexual abuse, mother-in-laws particularly involved in mental abuse and male family members involved in physical and mental abuse [25]. The available data were not sufficient for us to conduct further analyses of violence by type of perpetrator. Despite our interest in DV, the lack of available data meant that our pooled prevalence results relate mostly to IPV.

Our study has several limitations. Our estimates for prevalence and health outcomes have wide confidence intervals and high I^2^ statistics, reflecting relatively small pooled samples and high levels of between-study variance. Despite wide ranging prevalence estimates, similarities in study design justified pooling. Health outcome data varied in the definition of both violence exposure and health outcomes. Some studies only looked at exposure to exclusive violence categories (e.g. physical violence only); this produces a clearer picture of the association between the violence type and specific health outcome, but excludes the many women who are exposed to multiple types of violence. Some papers reported differing associations between different violence types and the relevant health outcome. Health outcomes were defined and measured using a range of methods. Most studies measured a limited range of health outcomes, for example reproductive health or mental health only. Combining data from these varied studies enabled the generation of pooled effect estimates but contributed to heterogeneity of the health outcome results.

Visual inspection of forest plots and attempts to look for patterns between outlying results and study characteristics were unproductive. We quantitatively assessed for sources of heterogeneity using univariate meta-regression. There were insufficient data to do this for all covariates for each analysis. Where it was performed, the small number of included studies limits reliability. None of the associations found were consistent across different analyses; they may represent false positives. As we were working with summary level data, we cannot infer that any associations found apply at the individual level.

Despite finding an association between exposure to violence and adverse health outcomes, causality cannot be inferred, as most studies were cross-sectional and few specified the temporal relationship between violence exposure and onset of the health problem. There was insufficient measurement of confounding variables and few studies adjusted for confounders. The associations demonstrated could be proxy for other causal relationships e.g. socioeconomic deprivation or level of education. Interestingly, when Al-Modallal adjusted for confounders, the positive crude odds ratios for suicidal thoughts and attempts became negative [27].

## Conclusions

Domestic violence research has progressed in the Arab region, but robust prevalence data are still required for some countries. Adopting standardised definitions and measurement tools would benefit the research field. However, definitions of violence need to retain meaning in different sociocultural contexts and adaptation of pre-existing tools may be necessary. Data on the frequency and severity of violence might elucidate mechanisms of impact. We also recommend further meta-analyses to synthesise data on the prevalence of IPV/DV in clinical settings in other geographical regions. To test causality between violence and adverse health outcomes, adequately powered longitudinal studies are needed with adjustment for confounding factors. A standardised approach to measuring the outcomes of domestic violence, including a core set of health outcomes with defined diagnostic criteria or tools, would enable comparison between studies and across different settings.

Many women accessing healthcare in the Arab region have been exposed to violence from family members or intimate partners. In some settings, the majority of women were affected. We have demonstrated that the adverse health outcomes of DV, well documented internationally, also affect Arab women. Our findings are of interest to clinicians and policy makers in the Arab region. Contact with a healthcare professional provides an opportunity to identify survivors of violence, offer support and refer to specialist services [69]. The WHO has called for a healthcare response to violence against women but much of the current evidence comes from high-income settings [70, 71]. The results from our review provide regional evidence which could be used to inform the development of healthcare interventions and policy. Interventions with an evidence base from high-income or other regional settings need local adaptation and evaluation, taking into account not only the cultural context but also the healthcare system resources and infrastructure and national policy and legal framework. We recommend further research in the Arab region to establish what a suitable healthcare response to domestic violence might look like.

## Additional files

Additional file 1: Search terms. (PDF 38 kb)

Additional file 2: Included studies summary table. (PDF 441 kb)

Additional file 3: Perpetrators of domestic violence. (PDF 117 kb)

Additional file 4: Additional forest plots. (PDF 81 kb)

Additional file 5: Summary of health outcome findings. (PDF 382 kb)

## Abbreviations

AAS: Abuse Assessment Screen
aOR: Adjusted odds ratio
CEDAW: Convention on the Elimination of all Forms of Discrimination Against Women
CI: Confidence interval
CTS-R: Conflict Tactics Scale, revised
DV: Domestic violence
EMR: Eastern Mediterranean Region
HITS: Hurt, Insult, Threaten and Scream
IPV: Intimate partner violence
NorAQ: NorVold Abuse Questionnaire
OR: Odds ratio
USPSTF: U.S. Preventative Services Task Force
VAW: Violence Against Women
WAST: Woman Abuse Screening Tool
WHO: World Health Organization
