# Supplementary material for: Prevalence and health outcomes of domestic violence amongst clinical populations in Arab countries: a systematic review and meta-analysis
Source: BMC Public Health. 2019 Mar 18;19:315. doi: 10.1186/s12889-019-6619-2 (PMC6421940; doi:10.1186/s12889-019-6619-2)
Supplement: Supplementary file 1 — Search terms. (PDF 38 kb) [file 12889_2019_6619_MOESM1_ESM.pdf]

<u>Additional file 1: Search terms for Medline via OvidSP</u>

These were adapted and modified for use on other databases.

Concept 1: Domestic violence

Domestic violence (MeSH)

Spouse abuse (MeSH)

Elder abuse (MeSH)

Intimate partner violence (MeSH)

Rape (MeSH)

Battered women (MeSH)

Human trafficking (MeSH)

Domestic violence.tw

Gender based violence.tw

Violence against women.tw

Intimate partner adj3 (violen* or abus*).mp

Wife beating.tw

(Batter* adj3 (wom?n OR wife OR wives OR mother* OR partner* OR wife OR spous*)).mp

(Abus* adj3 (wom?n OR wife OR wives OR mother* OR partner* OR wife OR spous*)).mp

((family OR household OR domestic OR relationship OR partner OR marital OR dating) adj3 (abus* OR violence OR victimi* OR batter*)).mp

Violence adj3 (partner* OR parent*).tw

((Abuse or violence) adj3 (emotional OR psychological OR physical OR sex*)).tw

Abusive relationship.tw

Survivor* of partner violence.tw

Sexual adj (abuse OR harassment).tw

Rape.tw

Sex* offen?es.tw

Forced adj (prostitution OR abortion OR sterilisation).tw

(early OR child OR forced) adj marriage.tw

Elder abuse.tw

Human trafficking.tw

Traffick* adj (wom*n OR sex* OR human).tw

Female Genital Mutilation.tw

Hono?r killing*.tw

Femicide.tw

Concept 2: Eastern Mediterranean countries

Eastern Mediterranean countr*.tw

Afghanistan.tw

Bahrain.tw

Djibouti.tw

Egypt.tw

Iran.tw

Iraq.tw

Jordan.tw

Kuwait.tw

Lebanon.tw

Libya.tw

Morocco.tw

Oman.tw

Pakistan.tw

Qatar.tw

Saudi Arabia.tw

Somalia.tw

Sudan.tw

Syria*.tw

Tunisia.tw

United Arab Emirates.tw

Yemen.tw

Palestin*.tw

Occupied Palestinian territory.tw

Israel.tw

Middle East.tw

arab countries.tw

gaza strip.tw

near east.tw

west bank.tw

MeSH terms:

Middle East

Afghanistan

Bahrain

Iran

Iraq

Israel

Jordan

Kuwait

Lebanon

Oman

Qatar

Saudi Arabia

Syria

United Arab Emirates

Yemen

Pakistan

Egypt

Libya

Morocco

Tunisia

Djibouti

Sudan

Somalia

Date limits:

2000 onwards only
