# Supplementary material for: Prevalence and health outcomes of domestic violence amongst clinical populations in Arab countries: a systematic review and meta-analysis
Source: BMC Public Health. 2019 Mar 18;19:315. doi: 10.1186/s12889-019-6619-2 (PMC6421940; doi:10.1186/s12889-019-6619-2)
Supplement: Supplementary file 2 — Included studies summary table. (PDF 441 kb) [file 12889_2019_6619_MOESM2_ESM.pdf]

Additional file 2

Author (year)	Country	Setting	Design	Recruitment	Response	Sample size	Survey type	Tool used for violence prevalence	Timescale	IPV or DV	Any violence prevalence +definition	Physical violence prevalence	Sexual violence prevalence	Emotional/ Psychological violence prevalence + definition	Control or Economic	Health impacts	AXIS quality
Abdelmegeid (2004)[22]	Egypt	MCHC centres 4 districts Cairo	CS	Clinics: R<br>Women: CON	-	1000	I	No – 12Qs violence behaviours	Lifetime	IPV	61%<br><br>P or S or E	36.9%	4.3%	14.7%<br><br>'Psychological'	-	-	Medium
Abdelhai and Mosleh (2005)[23]	Egypt	ANC at teaching hospital, Cairo	CS	Women: R	96%	376	I	HITS	Lifetime	IPV	30.6%<br><br>P or E	7.7%	-	17%<br><br>'Screamed or cursed frequently'	-	MH	Medium/ High
Abujilban et al (2015)[24]	Jordan	Postnatal department, MOH hospital	Case control	Hosptials: cluster random<br>Cases: consecutive<br>Controls: R	-	158	Q	WHO (physical)	Pregnancy	IPV	-	-	-	-	-	Reprod	Medium
Afifi et al (2011)[25]	Saudi Arabia	10 PHC clinics – 6 urban, 3 village, 1 Hejar	CS	Clinic: 2 stage proportionate cluster R<br>Women:CON	-	2000	I	WHO (modified)	Past month<br>Lifetime	DV	1m: 32.7%<br>LT: 39.3%<br><br>P or S or E	1m: 22.8%<br>LT: 17.9%	1m: 11.8%<br>LT: 6.9%	1m: 29.1%<br>LT: 35.9%<br><br>'Mental'	-	MH<br>Reprod<br>General	Medium
Ahmed and Elmari (2005)[26]	Sudan	Medical clinics – referrals from primary care	CS	Women: CON	87%	394	Q	No – 17Qs (based on Bradley et al 2002) no sexual Qs	12m	IPV<br><br>[DV]	41.6%<br><br>P or E	20.1%	-	30.2%<br><br>'Threat'	28.4%<br><br>'Control'	-	Medium/ High
Al Modallal (2012)[27] †	Jordan	UNWRA health centres for Palestinian refugees 3 cities	CS	Women: CON	-	300	Q	AAS	12m	IPV	43.4%<br><br>P or S or E	-	-	-	-	MH*	Medium
Al Modallal et al (2014)[28] †	Jordan	MOH and UNWRA health centres, 3 cities	CS	Women: CON	-	620	Q	AAS	12m	IPV	37.5% *<br><br>P or S or E	-	-	-	-	N/A	Medium
Al Modallal et al (2015)[29] †	Jordan	UNWRA health centres for Palestinian refugees	CS	Women: CON	-	300	Q	AAS: P + S<br>WHO: control<br>Other: E and Econ	Lifetime	IPV	78% *<br><br>P or S or E or Econ	22.7%	16.7%	50.3% *<br><br>'Emotional'	736.%<br>'Control'<br><br>50.3% *<br>'Econ'	-	Medium
Al Modallal (2016)[30] †	Jordan	UNWRA health centres for Palestinian refugees 3 cities	CS	Women: CON	72%	238	Q	AAS	12m	IPV	N/A	N/A	N/A	N/A	N/A	General*	Medium/ High

Additional file 2

Al Modallal (2017)[31] †	Jordan	MOH and UNWRA health centres, 3 cities	CS	Women: CON	-	709	Q	WHO (adapted)	Lifetime	IPV	-	51.5% *	24.5% *	-	68.8% *	-	Medium
Al-Nsour et al (2009)[32]	Jordan	PHC – 9 centres in Balka governate	CS	Clinic: R<br>Women: CON	98%	356	Q	No – single open ended question	12m	IPV	87%<br><br>P or E	19.6%	-	47.5% ‘Emotional’<br><br>[12.3% ‘neglect’]	-	-	Medium/ High
Al-Serkal et al (2014)[33]	UAE	Primary healthcare centre in Dubai	CS	Women: R	-	700	I	WHO	12m<br>Lifetime	IPV	-	12m: 7.1%<br>LT: 31%	12m: 3.7%<br>LT: 22%	12m: 7.5%<br>LT: 40.9%<br><br>‘Psychological’	-	MH<br>Reprod<br>General	Medium
Al Shdayfat (2017a)[34] ‡	Jordan	MCHC – 3 urban, 2 rural. Syrian refugees.	CS	Clinics: purposive<br>Women: CON	73%	182	Q	NORAQ (Arabic)	12m<br>Lifetime	DV	-	-	-	12m: 44.5%<br>LT: 51.6%<br><br>‘Emotional’	-	MH*	Medium/ High
Al Shdayfat (2017b)[35] ‡	Jordan	MCHC – 3 urban, 2 rural. Syrian refugees.	CS	Clinics: purposive<br>Women: CON	73%	182	Q	NORAQ (Arabic)	12m<br>Lifetime	DV	-	12m:14.8%<br>LT: 31.3%*	-	-	-	MH	Medium
Alzahrani et al (2016)[36]	Saudi Arabia	Primary Healthcare Centres, Taif	CS	2 stage probability sampling	99%	497	Q	HITS	12m	IPV	11.9%<br><br>P or E	-	-	-	-	-	Medium
Anes Jellali et al (2014)[37] §	Tunisia	Family planning centre, Monastir	CS	Women: R	-	197	I	WAST + econ Q	Lifetime	IPV	56.9% *<br><br>P or S or E or Econ	32% *	10.6% *	56.9% *<br><br>‘Psychological’	41.1% *<br><br>‘Economic’	MH*<br>Reprod*	Medium
Anes Jellali et al (2015)[38] [FRENCH] §	Tunisia	Family planning centre, Monastir	CS	Women: R	-	197	I	WAST + econ Q	Lifetime	IPV	56.9%<br><br>P or S or E or Econ	32%	10.6%	56.9%<br><br>‘Psychological’	41.1%<br><br>‘Economic’	Reprod	Medium
Awwad et al (2014)[39]	Lebanon	Outpatient gynae clinic, University Hospital	CS	Women: CON	91%	91	I	No – 11Qs appropriate questions	Lifetime	IPV	-	40.6%	33%	64.8%‘Verbal’*<br><br>[19% Emotional 22% Social]	33%<br><br>‘Economic’	-	Medium/ High
Azm et al (2009)[40]	Saudi Arabia	Antenatal, IP ante/postnatal, Emergency O+G, CHC	CS	Women: CON	88%	350	I	No – 32Qs questionnaire designed	Lifetime<br>Pregnancy	IPV	LT: 78%<br>-<br>P or S or E or Econ	LT: 78%<br>PG: 54%	LT: 48.3%<br>-	LT: 72.9%<br>-<br>‘Psychological’	LT: 32.6%<br>-<br>‘Financial’	MH<br>Reprod	Medium
Bakr and Ismail (2005)[41]	Egypt	University hospital - varied outpatient clinics	CS	Women: CON	-	509	I	No – 21Qs (Bradley et al 2002 modified)	Lifetime	IPV	89.8%<br><br>P or S or E	N/A	17.1%	47.9%<br><br>‘Threat’	88.4%<br><br>‘Control’	General	Medium
Barnawi (2017)[42]	Saudi Arabia	Primary health care centre, Riyadh	CS	Women: CON	95%	720	Q	No – 14 behaviours questionnaire designed	12m	DV	20%<br><br>P or S or E	4%	2.1%	13.9% ‘Emotional’<br><br>(6.8% social)	5.3%<br><br>‘Economic’	General	Medium/ High

Additional file 2

								from literature									
Boufettal et al (2012)[43] [FRENCH]	Morocco	University hospital – ANC and inpatient	CS	Not stated – convenience?	89%	867	I	No – 9 violence behaviours listed	Pregnancy	IPV	12.3%<br><br>P or S	-	1.2%	-	-	MH Reprod	Medium
Clark et al (2008)[44] ¶	Jordan	Family planning, 7 clinics	CS	Systematic prob proportionate to size	70%	353	Q	WHO (modified)	Lifetime	IPV	-	31%	20%	-	-	Reprod	Medium/ High
Clark et al (2009a)[45] ¶	Jordan	Family planning, 7 clinics	CS	Systematic prob proportionate to size	70%	517	Q	WHO (modified)	Lifetime	IPV	-	31.2% *	18.8% *	73.4% *<br><br>'Psychological'	97.2% *<br><br>'Control'	-	Medium/ High
Clark et al (2009b)[46] ¶	Jordan	Family planning, 7 clinics	CS	Systematic prob proportionate to size	55%	390	Q	WHO (modified)	Pregnancy	DV	-	15.4% *	-	-	-	-	Medium
Clark et al (2010)[47] ¶	Jordan	Family planning, 7 clinics	CS	Systematic prob proportionate to size	70%	418	Q	WHO (modified)	Lifetime	IPV<br><br>[DV]	38% *<br><br>P or S	29%	18%	-	-	-	Medium/ High
Daoud et al (2017)[48]	Israel (Arabs only)	Maternal and child health clinics- 21 in Arab localities	CS	Clinic: stratified proportional cluster<br>Women: CON	76%	436	I	US Preventative Services Task Force Family screening tool for IPV	Lifetime	IPV	66.7%<br><br>P or S or E or Econ	10.6%	-	49.7%<br><br>'Verbal or emotional'	-	-	High
Eldoseri et al (2014)[49] ‖	Saudi Arabia	Primary healthcare, 6 clinics.	CS	Clinic: purposive<br>Women: CON	94%	200	I	WHO (modified) physical only	12m<br>Lifetime	IPV	-	12m: 16%<br>LT: 44.5%	-	-	-	MH General	Medium/ High
Eldoseri and Sharps (2017)[50] ‖	Saudi Arabia	Primary healthcare, 6 clinics.	CS	Clinic: purposive<br>Women: CON	94%	200	I	WHO (modified) physical only	12m<br>Lifetime	IPV	-	12m: 16%*<br>LT: 44.5%*	-	-	-	-	High
Haddad et al (2011)[51]	Jordan	Maternal and child healthcare centres – urban and rural	CS	Women: CON	98%	175	Q	NORAQ (Arabic – validated by this study)	Lifetime	DV	-	30%	6%	39%<br><br>'Emotional'	-	MH	Medium/ High
Hammoury and Khawaja (2007)[52] ††	Lebanon	ANC in UNWRA clinic – refugees from Palestine	CS	Women: CON	99%	349	I	AAS (sexual abuse Q modified)	12m<br>Lifetime<br>Pregnancy<br><br>(pregnant women only)	DV	-<br>LT: 68.8%<br>-<br><br>P or S or E *	12m:19.2%<br>LT: 59.3%<br>PG: 11.5%<br><br>*	12m:26.4%<br>-<br>-<br><br>*	12m: 16.1%<br>-<br>-<br><br>'Fear of husband' *	-	-	Medium
Hammoury et al (2009)[53]	Lebanon	ANC in UNWRA clinic – refugees from	CS	Women: CON	99%	349	I	AAS (sexual abuse Q	12m<br>Lifetime<br>Pregnancy	DV	-<br>LT: 59%<br>-	12m:19.2%<br>-<br>PG: 11.5%	-	-	-	Reprod*	Medium/ High

Additional file 2

††		Palestine						modified)	(pregnant women only)		P or E						
Ibrahim et al (2015)[54]	Egypt	Obstetric outpatient unit – university hospital	Prospective cohort	Women: CON	85%	1857	I	NORAQ (Arabic)	Pregnancy	IPV	44.1%<br><br>P or S or E	15.9%	10%	32.6%<br><br>'Emotional'	-	Reprod	Medium/ High
Khawaja and Hammoury (2008)[55] ††	Lebanon	ANC in UNWRA clinic – refugees from Palestine	CS	Women: CON	99%	349	I	AAS (sexual abuse Q modified)	12m<br>Pregnancy<br><br>(pregnant women only)	IPV	-	12m:19.2%<br>PG: 11.4%	12m: 26.4%<br>-	12m: 16.1%<br>-<br><br>'Fear of husband'	-	Reprod	Medium
Malik et al (2017)[56]	Iraq	Public primary healthcare centre – for reproductive problems	CS	Women: R	82%	82	I	WHO (adapted)	12m	IPV	72%<br><br>P or S or E	39%	18.3%	61%<br><br>'Emotional'	-	-	Medium
Mamdouh et al (2012)[57]	Egypt	Family health centres – 12 centres, urban and rural	CS	Clinics: R<br>Women: systematic	93%	3271	I	No 26Qs (designed questionnaire from literature)	Lifetime	IPV	77%<br><br>P or S or E or Econ	50.2%	37.1%	71%<br><br>'Emotional'	40.8%<br><br>'Economic'	-	High
Okour and Badarneh (2011)[58]	Jordan	Antenatal – MCH clinics 2 rural, 2 urban. Bedouin community.	CS	? sampling	97%	303	I	WHO	Pregnancy	IPV	40.9%<br><br>P or S or E	34.6%	15.5%	28.1%<br><br>'Psychological'	-	-	Medium/ High
Oweis et al (2010)[59]	Jordan	Maternal and Child Health Centres, 5 in Irbid City.	CS	Women: CON	83%	316	Q	No (5 Y/N questions)	Pregnancy	IPV<br><br>[DV]	-	10.4%	5.7%	23.7%<br><br>'Verbal'<br><br>('Emotional' 23.4%)	-	MH<br>Reprod	Medium/ High
Spencer et al (2015)[60]	Jordan and Lebanon	Primary care clinics–Syrian national and Palestinian refugees from Syria	CS	Women: CON	-	385	I	No – survey developed and tested by researchers	Since arriving in Jordan/ Lebanon + pregnancy	DV	-<br>-<br>-	Jor: 7.5%<br>Leb: 6%<br>PG: 6.3%	Jor: 1.5%<br>Leb: 0%<br>-	Jor: 6%<br>Leb: 6.5%<br>-<br><br>'Emotional'	Jor: 0%<br>Leb: 0%<br>-<br><br>'Economic'	Reprod	Medium
Tashkandi and Rasheed	Saudi Arabia	Primary care – 16 clinics Medina City	CS	Clinics: simple R<br>Women: CON	68%	689	I	CTS-R (CTS-revised +	12m<br>Lifetime	IPV	12m:58.5%<br>LT:57.8%	12m:25.7%<br>LT: 26.9%	-	12m: 57.5%<br>-	-	-	Medium/ High

Additional file 2

(2009)[61]								added Qs)			P or E			'Emotional'			
Usta et al (2007)[62]	Lebanon	Primary care clinics, 4 clinics	CS	Women: CON	90% estim ate	1415	I	No 4Qs (based on Haj-Yahia and adapted)	Lifetime	DV	35%<br><br>P or E or Econ	23%	-	32%<br><br>'Insults and threats'	12%<br><br>'Economic'	MH<br>Reprod<br>General	Medium
**Summary of study design and details**<br>**=**<br>**Total**<br>**29 studies**<br><br>**(41 papers)**	Egypt 5<br>Iraq 1<br>Israel (Arabs) 1<br>Jordan 8<br>Lebanon 3<br>Jor+Leb 1<br>Morocco 1<br>Saudi Arabia 6<br>Sudan 1<br>Tunisia 1<br>UAE 1	Community 21<br>Hospital 7<br>Both 1<br>———<br>Women's health setting 16<br>General health setting 13<br>———<br>Refugee population 4				Sam ple size:<br><br>min 82<br><br>max 3271	I:19<br>Q:10	Validated tool: 17<br>*WHO 7*<br>*NORAQ 3*<br>*AAS 2*<br>*HITS 2*<br>*WAST 1*<br>*CTS-R 1*<br>*USPSTF 1*<br><br>Non-validated tool: 12		IPV 19<br><br>DV 6<br><br>Both 4	Any violence: 21 studies<br><br>P+S+E 13<br>P+S 2<br>P+E 6	Physical violence: 25 studies	Sexual violence: 19 studies	Emotional and/or psychological violence: 25 studies	Control violence: 4 studies<br><br>Economic violence: 8 studies	Health outcome: 19 studies<br><br>MH: 12<br>Reprod: 12<br>General: 7	

Key: CS = cross sectional, R = random, CON = convenience, I= interview, Q=questionnaire, 12m= 12 months, PG=Pregnancy, LT= lifetime, IPV = intimate partner violence, DV = domestic violence, [DV] = paper also looked at DV but main results presented are for IPV, P = physical, S = sexual, E = emotional/psychological, Econ=economic, Jor= Jordan, Leb=Lebanon, MH= mental health, Reprod= reproductive health, General = general medical or health, N/A= data not appropriate/not used

* prevalence numbers taken from this paper if multiple papers from same study

† multiple papers from same study

‡ multiple papers from same study

§ multiple papers from same study

¶ multiple papers from same study

‖ multiple papers from same study

†† multiple papers from same study
