# Supplementary material for: Prevalence and health outcomes of domestic violence amongst clinical populations in Arab countries: a systematic review and meta-analysis
Source: BMC Public Health. 2019 Mar 18;19:315. doi: 10.1186/s12889-019-6619-2 (PMC6421940; doi:10.1186/s12889-019-6619-2)
Supplement: Supplementary file 3 — Perpetrators of domestic violence. (PDF 117 kb) [file 12889_2019_6619_MOESM3_ESM.pdf]

Appendix 3: Perpetrators of domestic violence

Paper	Violence type	Husband	Husband + other	Any family non-husband	Brother	Mother	Father	Mother-in-law	Father-in-law	Other	Comments
Afifi et al (2011)[25]	Physical	59.2	8.1		11.7		6.4	2.8	0.6	19.9	
	Mental	45.9	9.3		7.3		7.7	10.6	2.6	22.2	
	Sexual	85.4	0.7		0.7		0.7		1.5	8.7	
Ahmed and Elmari (2005)[26]	Control, threat or physical			25.6							
Al-Shdayfat (2017a)[34]	Emotional	21.4	19.8		13					Other family member 12	
Al-Shdayfat (2017b)[35]	Physical	38.6			10		14				
Barnawi (2017)[42]	All types	81.2					Wife's family 11.1		Husband's family 6.9	Other wife 4.9	
Clark et al (2009b)[46]	Physical during pregnancy	83.3	8.3				Natal family 5		Husband's family 1.7	Someone else 1.7	
Clark et al (2010)[47]	Physical and sexual	-	-	100	1	1	0	30	9	Sister-in-law 27<br>Brother-in-law 17<br>Other-in-law 8	Percentages relate to violence experienced by family perpetrators, not including husband
Haddad et al (2011)[51]	Emotional	Husband and ex-husband 71			30						
Oweis et al (2010)[59]	Physical<br>Emotional<br>Verbal			1.9<br>11.1<br>13.9				Most perpetrators were mother-in-law			Prevalence of violence by family member experienced amongst all survey respondents
Spencer et al (2015)[60]	Physical during pregnancy	73		45							
Usta et al (2007)[62]	Verbal, emotional, physical or economic	65	More than one offender 13			Mother or mother-in-law 8		Mother or mother-in-law 8		Own parents/ male family 18	

Note: Unless otherwise specified, percentages relate to the percentage of abused women experiencing domestic violence committed by different perpetrators (husband or other family members)
