# Supplementary material for: Prevalence and health outcomes of domestic violence amongst clinical populations in Arab countries: a systematic review and meta-analysis
Source: BMC Public Health. 2019 Mar 18;19:315. doi: 10.1186/s12889-019-6619-2 (PMC6421940; doi:10.1186/s12889-019-6619-2)

Additional file 4 – forest plots for additional analyses

12-month physical IPV:

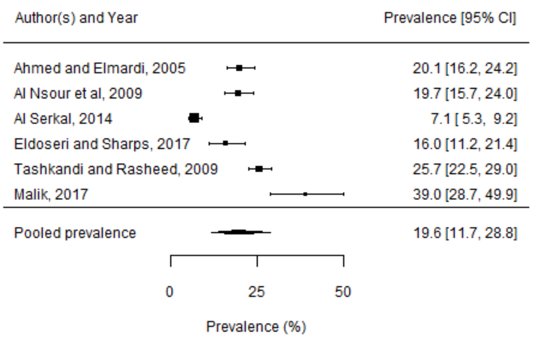

12-month emotional/psychological IPV:

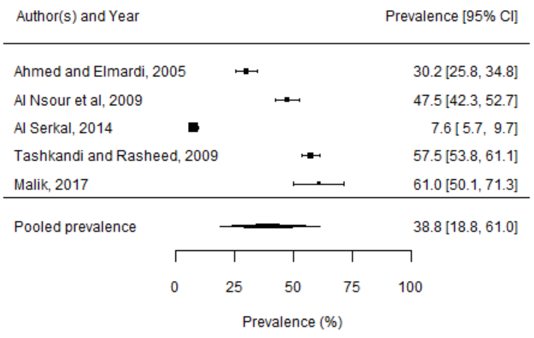

Lifetime physical DV:

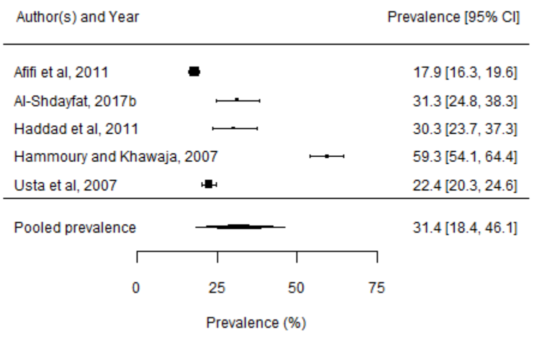

Lifetime economic IPV:

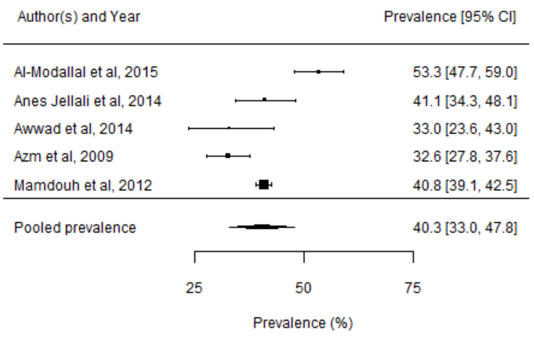

Supplement: Supplementary file 4 — Additional forest plots. (PDF 81 kb) [file 12889_2019_6619_MOESM4_ESM.pdf]
