# Supplementary material for: Prevalence and health outcomes of domestic violence amongst clinical populations in Arab countries: a systematic review and meta-analysis
Source: BMC Public Health. 2019 Mar 18;19:315. doi: 10.1186/s12889-019-6619-2 (PMC6421940; doi:10.1186/s12889-019-6619-2)
Supplement: Supplementary file 5 — Summary of health outcome findings. (PDF 382 kb) [file 12889_2019_6619_MOESM5_ESM.pdf]

# Additional file 5: Health outcomes as presented by primary studies

Results are presented in the table below for health conditions where data was available from at least 3 studies. Reported results are rounded to 1 decimal point or 2 sf.

## MENTAL HEALTH

### Depression

| Paper | Perp | Timescale | Outcomes | Diagnostic method/tool | Violence type | Statistics (significant in bold)<br>OR, p value, other |
|---|---|---|---|---|---|---|
| Abdelhai and Mosleh (2015)[23] # | IPV | Lifetime | Depression (only without anxiety) | HADS | Any | **OR 3.1 (1.1-8.3), p 0.03** |
| Al-Shdayfat (2017a)[34] | IPV | 12m/lifetime? | Depression | Not documented | Emotional | **$X^2$22.2, p 0.001** |
| Al-Shdayfat (2017b)[35] | DV | Lifetime? | Depression | Not documented | Physical | **$X^2$4.5, p value 0.034** |
| Anes Jellali et al (2014)[37] # | IPV | Lifetime | Depression | HADS score 8 | Any | **OR 3.5 (1.3-9.7), p 0.012** |
| Bakr and Ismail (2005)[41] | IPV | Lifetime | Depression | Participant reported | Control | **p <0.05** |
| Boufettal et al (2012)[43] # | IPV | Pregnancy | Depression | Not documented | Physical and/or sexual | **p < 0.0005** |
| Eldoseri et al (2014)[49] # | IPV | Lifetime | Antidepressant use in the last 4 weeks | Participant reported. WHO Violence against Women questionnaire (version 10.0) – adapted version | Physical | **Reported: OR 17.4 (2-152)**<br>**Calculated: OR 12.4 (1.5-99.7)**<br>**p= 0.01** |
| Haddad et al (2011)[51] | DV | Lifetime | Depression | 'depression feelings' participant reported | Any: physical/sex/emot #<br>Physical<br>Sexual<br>Emotional | **r= 0.42, p <0.001**<br>**r= 0.36, p<0.001**<br>**r=0.16, p<0.001**<br>**r=0.41, p<0.001** |

# Included in meta-analysis

### Suicidal thoughts

| Paper | Perp | Timescale | Outcomes | Diagnostic method/tool | Violence type | Statistics (significant in bold)<br>OR, p value, other |
|---|---|---|---|---|---|---|
| Al-Modallal (2012)[27] | IPV | 12m | Suicidal thoughts | Participant reported | Any | Crude OR 11.94 (4.5-31.6)<br>**Adjusted OR 0.04 (0.009-0.15)** |
| Al-Serkal et al (2014)[33] | IPV | Lifetime | Suicidal thoughts | Not documented | Physical ONLY<br>Sexual ONLY<br>Psychological ONLY | **OR 5.1 (3.2-8.1), p=0.001**<br>**OR 4.7 (2.9-7.5), p=0.001**<br>**OR 5.8 (3.3-10.2), p=0.001**<br>[stepwise logistic regression] |
| Eldoseri et al (2014)[49] | IPV | Lifetime | Suicidal thoughts | Participant reported. WHO Violence against Women questionnaire (version 10.0) – adapted version | Physical | **OR 6.6 (2.4-17.9)**<br>**p<0.001** |

| | | | | | | |
|---|---|---|---|---|---|---|
| Usta et al (2007)[62] | DV | Lifetime | Suicidal thoughts | Participant reported | Any | **ANOVA, p=0.000** |

## Suicide attempts

| Paper | Perp | Timescale | Outcomes | Diagnostic method/tool | Violence type | Statistics (significant in bold)<br>OR, p value, other |
|---|---|---|---|---|---|---|
| Al-Modallal (2012)[27] | IPV | 12m | Suicide attempts | Participant reported | Any | Crude OR 9.1 (2.6-31.5)<br>**Adjusted OR 0.02 (0.002-0.19)** |
| Al-Serkal et al (2014)[33] | IPV | Lifetime | Suicide attempts | Not documented | Physical ONLY<br>Sexual ONLY<br>Psychological ONLY | **OR 8.8 (4.7-16.3), p=0.001**<br>**OR 5.2 (2.8-9.4), p=0.001**<br>**OR 5.1 (2.5-10.3), p=0.001**<br>[stepwise logistic regression] |
| Boufettal et al (2012)[43] | IPV | Pregnancy | Suicide attempts | Not documented | Physical and/or sexual | **p<0.002** |
| Eldoseri et al (2014)[49] | IPV | Lifetime | Suicide attempts | Participant reported. WHO Violence against Women questionnaire (version 10.0) – adapted version | Physical | OR 4 (1-16.1)<br>p=0.06 |

## Sleep

| Paper | Perp | Timescale | Outcomes | Diagnostic method/tool | Violence type | Statistics (significant in bold)<br>OR, p value, other |
|---|---|---|---|---|---|---|
| Al-Modallal (2012)[27] # | IPV | 12m | Sleep | Epworth Sleepiness Scale | Any | Crude OR 1.1 (0.7-1.8)<br>Adjusted OR 0.64 (0.4-1.1)<br>p=0.72 |
| Al-Shdayfat (2017a)[34] | DV | Lifetime | Insomnia | Participant reported | Psych | **p=0.001** |
| Al-Shdayfat (2017b)[35] | DV | Lifetime | Insomnia | Participant reported | Physical | p= non-significant |
| Bakr and Ismail (2005)[41] # | IPV | Lifetime | Insomnia | Participant reported | Control | **p<0.05** |
| Boufettal et al (2012)[43] # | IPV | Pregnancy | Sleep problems | Not documented | Any: physical or sexual | **p<0.0005** |
| Eldoseri et al (2014)[49] # | IPV | Lifetime | Taken sleep or relaxing medication in the last 4 weeks | Participant reported. WHO Violence against Women questionnaire (version 10.0) – adapted version | Physical | Reported: OR 2.1 (0.7-6.6)<br>Calculated: OR 1.7 (0.5-5.2)<br>p=0.2 |
| Haddad et al (2011)[51] | DV | Lifetime | Insomnia | Not documented | Any #<br>Physical<br>Emotional | **r=0.43, p<0.001**<br>**r=0.37, p<0.001**<br>**r=0.45, p<0.001**<br>[correlation coefficient] |
| Usta et al (2007)[62] # | DV | Lifetime | Insomnia | Participant reported | Any | **ANOVA, p=0.000** |

# Included in meta-analysis

## Memory

| Paper | Perp | Timescale | Outcomes | Diagnostic method/tool | Violence type | Statistics (significant in bold) OR, p value, other |
|---|---|---|---|---|---|---|
| Al-Serkal et al (2014)[33] | IPV | Lifetime | Some/many or extreme memory or concentration problems | Not documented | Physical ONLY | **OR 5.2 (3.3-8.8)** |
| Eldoseri et al (2014)[49] | IPV | Lifetime | Some/many or extreme memory or concentration problems in the last 4 weeks | Participant reported. WHO Violence against Women questionnaire (version 10.0) – adapted version | Physical | OR 1.8 (0.9-3.2)<br>p=0.07 |
| Usta et al (2007)[62] | DV | Lifetime | Forgetfulness | Participant reported | Any | **p=0.000 (ANOVA)** |

**Other mental problems with significant associations found by studies:** anxiety,[37] anxiety and depression,[23,37] postnatal depression,[40] stress,[25,59] distress/anguish.[33,51]

**Other mental health problems with no evidence of significant associations found by studies:** anxiety,[23] postnatal depression.[40]

---

# REPRODUCTIVE HEALTH

## Vaginal bleeding

| Paper | Perp | Timescale | Outcomes | Diagnostic method/tool | Violence type | Statistics (significant in bold) OR, p value, other |
|---|---|---|---|---|---|---|
| Afifi et al (2011)[25] | DV | Lifetime | Vaginal bleeding last 4w | Participant reported | Any | **OR 1.8 (1.3-2.5)**<br>**p=0.000** |
| Bakr and Ismail (2005)[41] | IPV | Lifetime | Irregular period | ? | Control | Non-significant |
| Usta et al (2007)[62] | DV | Lifetime | Menstrual irregularities | Participant reported | Any | **p=0.000 (ANOVA)** |

## Unplanned pregnancy

| Paper | Perp | Timescale | Outcomes | Diagnostic method/tool | Violence type | Statistics (significant in bold) OR, p value, other |
|---|---|---|---|---|---|---|
| Azm et al (2009)[40] | IPV | Lifetime | Unplanned pregnancy | Participant reported | Any<br>Physical<br>Sexual<br>Emotional/psych | p= 0.095<br>p=0.032<br>**p=0.008**<br>**p=0.007** |

| | | | | | | |
|---|---|---|---|---|---|---|
| Hammoury and Khawaja (2007)[52] | DV | During pregnancy | Undesired pregnancy | Participant reported | Any: emotional or physical | OR 1.6 (0.66-3.8) |
| Ibrahim et al (2015)[54] | IPV | Pregnancy | Unwanted pregnancy | Not documented | Any | **OR 6.4 (4.9-8.4)** |
| Khawaja and Hammoury (2008)[55] | IPV | 12m | Undesired pregnancy | Not documented | Sexual | AOR 1.23 (0.72-2.1) |
| Oweis et al (2010)[59] | IPV | Pregnancy | Unplanned pregnancy | Participant reported | Any | **OR 2.5 (1.4-4.4)**<br>**p= 0.00** |

## Abortion

| **Paper** | **Perp** | **Timescale** | **Outcomes** | **Diagnostic method/tool** | **Violence type** | **Statistics (significant in bold)**<br>**OR, p value, other** |
|---|---|---|---|---|---|---|
| Afifi et al (2011)[25] # | DV | Lifetime | Abortion | Participant reported | Any | **OR 1.3 (1.1-1.6)**<br>**p=0.006** |
| Anes Jellali et al (2015)[38] | | Lifetime | Induced abortion | Not documented | Any<br>Physical<br>Sexual<br>Psychological<br>Economic | **p<0.0001**<br>**p<0.001**<br>**p<0.03**<br>**p<0.0001**<br>**p<0.0001** |
| Azm et al (2009)[40] # | IPV | Lifetime | No vs one vs multiple abortions | Participant reported | Any<br>Physical<br>Sexual<br>Psychological | **p=0.003**<br>**p=0.002**<br>**p=0.003**<br>p=0.245 |
| Boufettal et al (2012)[43] # | IPV | Pregnancy | Abortion | Not documented | Any: physical or sexual | **p<0.0005** |
| Ibrahim et al (2015)[54] # | IPV | Pregnancy | Complete abortion | Medical records | Any<br>Physical | **RR 5.4 (2.4-12.3)**<br>**RR 10.9 (5.6-21.5)** |
| Spencer et al (2015)[60] # | DV | Since coming to Jordan/ Lebanon | Abortion | Not documented | Any | Absolute numbers and % only reported by paper.<br>Calculated: OR 0.94 (0.17-6.3) |
| Usta et al (2007)[62] # | DV | Lifetime | Abortion | Participant reported | Any | **p=0.003 (ANOVA)** |

# Included in meta-analysis

## Premature labour

| **Paper** | **Perp** | **Timescale** | **Outcomes** | **Diagnostic method/tool** | **Violence type** | **Statistics (significant in bold)**<br>**OR, p value, other** |
|---|---|---|---|---|---|---|

| | | | | | | |
|---|---|---|---|---|---|---|
| Azm et al (2009)[40] | IPV | Lifetime | Premature and LBW | Participant reported | Any<br><br>Physical<br>Sexual | **p=0.0004** (for all postnatal complications)<br>**p=0.006**<br>**p=0.004** |
| Abujilban et al (2015)[24] | IPV | Pregnancy | Preterm birth | Medical records | Physical | p=0.8 |
| Ibrahim et al (2015)[54] | IPV | Pregnancy | Preterm labour | Medical records | Any<br>Physical | **RR 2.4 (1.5-3.8)**<br>**RR 5.7 (37.-8.8)** |

**Other reproductive health problems with significant associations found by studies:** vaginal discharge,[33,62] not using contraception,[40,44] stillbirth,[54] postpartum haemorrhage,[40] low birthweight,[24,54] neonatal death,[54] threatened abortion,[54] placental separation,[43] PROM,[54] fetal distress.[54]

**Other reproductive health problems with no evidence of significant associations found by studies**: not using contraception,[40] stillbirth,[43] antepartum haemorrhage,[54] postpartum haemorrhage,[40,54] PROM,[43] retroplacental haematoma,[43] assisted newborn ventilation,[24] caesarean delivery.[54]

---

## MEDICAL

### Dizziness

| **Paper** | **Perp** | **Timescale** | **Outcomes** | **Diagnostic method/tool** | **Violence type** | **Statistics (significant in bold)**<br>**OR, p value, other** |
|---|---|---|---|---|---|---|
| Afifi et al (2011)[25] | DV | Lifetime | Dizziness in last 4 weeks | Participant reported | Any | **OR 1.9 (1.6-2.3)**<br>**p=0.000** |
| Al-Modallal (2016)[30] | IPV | 12m | Recurrent dizziness last year | Participant reported | Physical ONLY<br>Sexual ONLY<br>Psychological ONLY | **P<0.01**<br>**P<0.05**<br>**P=0.009** |
| Al-Serkal et al (2014)[33] | IPV | Lifetime | Dizziness | Not documented | Physical ONLY | **OR 3.1 (2.1-4.5)** |
| Usta et al (2007)[62] | DV | Lifetime | Dizziness | Participant reported | Any | **p=0.000 (ANOVA)** |

### Pain

| **Paper** | **Perp** | **Timescale** | **Outcomes** | **Diagnostic method/tool** | **Violence type** | **Statistics (significant in bold)**<br>**OR, p value, other** |
|---|---|---|---|---|---|---|
| Afifi et al (2011)[25] # | DV | Lifetime | Pain in last 4 weeks by severity | Participant reported | Any | **Mild OR 1.8 (1.4-2.3)**<br>**Moderate OR 3.9 (3-5.1)**<br>**Severe OR 6.3 (4.0-9.7) #**<br>**Very severe OR 8.9 (3.1-25)**<br>**p=0.000** |

| | | | | | | |
|---|---|---|---|---|---|---|
| Al-Modallal (2016)[30] # | IPV | 12m | Back pain #<br><br><br><br>Joint pain<br><br><br><br>Fibromyalgia | Participant reported | Physical ONLY #<br>Sexual ONLY<br>Psych ONLY<br><br>Physical ONLY<br>Sexual ONLY<br>Psych ONLY<br><br>Physical ONLY<br>Sexual ONLY<br>Psych ONLY | Non-significant<br>Non-significant<br>**p<0.01**<br><br>Non-significant<br>Non-significant<br>**p=0.004**<br><br>Non-significant<br>**p<0.05**<br>**p=0.007** |
| Al-Serkal et al (2014)[33] | IPV | Lifetime | Mod/severe/ extreme pain | Not documented | Physical AND sexual #<br>Physical ONLY | **OR 3.5 (1.9-6.2)**<br>**OR 3.3 (1.9-5.9)** |
| Bakr and Ismail (2005)[41] # | IPV | Lifetime | Bone ache | Participant reported | Control | **p<0.5** |
| Eldoseri et al (2014)[49] # | IPV | Lifetime | Pain or discomfort last 4 weeks | Participant reported. WHO Violence against Women questionnaire (version 10.0) – adapted version | Physical | **OR 2.2 (1.2-3.9)**<br>**p=0.007** |
| Usta et al (2007)[62] # | DV | Lifetime | Backache<br>Arthralgia<br>Abdo pain | Participant reported | Any | **p=0.000 (ANOVA)**<br>**p=0.000 (ANOVA)**<br>**p=0.000 (ANOVA)** |

# Included in meta-analysis

## Hypertension

| Paper | Perp | Timescale | Outcomes | Diagnostic method/tool | Violence type | Statistics (significant in bold)<br>OR, p value, other |
|---|---|---|---|---|---|---|
| Afifi et al (2011)[25] # | DV | Lifetime | Hypertension | BP measurement | Any | OR 1.23 (0.92-1.7)<br>p=0.144 |
| Al-Modallal (2016)[30] # | IPV | 12m | Hypertension | Participant reported | Physical ONLY #<br>Sexual ONLY<br>Psychological ONLY | Non-significant<br>**p<0.05**<br>Non-significant |
| Bakr and Ismail (2005)[41] # | IPV | Lifetime | Hypertension | Participant reported | Control | Non-significant |
| Barnawi (2017)[42] # | DV | 12m | Hypertension | Not documented | Any | **p=0.01** |
| Usta et al (2007)[62] # | DV | Lifetime | Hypertension | Participant reported | Any | p=0.006 (ANOVA) |

# Included in meta-analysis

## Diabetes

| Paper | Perp | Timescale | Outcomes | Diagnostic method/tool | Violence type | Statistics (significant in bold)<br>**OR, p value, other** |
|---|---|---|---|---|---|---|
| Al-Modallal (2016)[30] | IPV | 12m | Diabetes | Participant reported | Physical ONLY<br>Sexual ONLY<br>Psychological ONLY | Non-significant<br>Non-significant<br>Non-significant |
| Bakr and Ismail (2005)[41] | IPV | Lifetime | Diabetes | Participant reported | Control | Non-significant |
| Barnawi (2017)[42] | DV | 12m | Diabetes | Not documented | Any | **p=0.027** |

## General health

| Paper | Perp | Timescale | Outcomes | Diagnostic method/tool | Violence type | Statistics (significant in bold)<br>**OR, p value, other** |
|---|---|---|---|---|---|---|
| Afifi et al (2011)[25] | DV | Lifetime | Perceived general health | Participant reported | Any | **Reasonable OR 0.36 (0.21-0.63)**<br>**Good OR 0.23 (0.13-0.39)**<br>**Excellent OR 0.15 (0.084-0.26)**<br>**p=0.000** |
| Al-Modallal (2016)[30] | IPV | 12m | General health | Participant reported | Physical ONLY | Non-significant |
| Al-Serkal et al (2014)[33] | IPV | Lifetime | Self reported poor or very poor health | Not documented | Physical AND sexual<br>Physical ONLY | **OR 7.1 (2.3-21.9)**<br>**OR 6.4 (1.9-21.2)** |
| Eldoseri et al (2014)[49] | IPV | Lifetime | Good/ excellent health status | Participant reported. WHO Violence against Women questionnaire (version 10.0) – adapted version | Physical | **OR 0.5 (0.3-0.9)**<br>**p=0.02** |

## Daily activities

| Paper | Perp | Timescale | Outcomes | Diagnostic method/tool | Violence type | Statistics (significant in bold)<br>**OR, p value, other** |
|---|---|---|---|---|---|---|
| Afifi et al (2011)[25] | DV | Lifetime | Problems in daily activities in the last 4 weeks | Participant reported | Any | **Few OR 1.8 (1.4-2.2)**<br>**Some OR 2.6 (2.0-3.3)**<br>**Many OR 3.7 (2.5-5.6)**<br>**Unable to do it OR 4.1 (0.98-17.4)**<br>**p=0.000** |
| Al-Serkal et al (2014)[33] | IPV | Lifetime | Some/many problems or unable to perform usual activities | Not documented | Physical AND sexual<br>Physical ONLY | **OR 5.4 (2.8-10.5)**<br>**OR 4.3 (2.1-8.7)** |

| | | | | | | |
|---|---|---|---|---|---|---|
| Eldoseri et al (2014)[49] | IPV | Lifetime | Problems with daily functions in the last 4 weeks | Participant reported. WHO Violence against Women questionnaire (version 10.0) – adapted version | Physical | OR 1.0 (0.5-2.0)<br>p=0.9 |

## Mobility

| Paper | Perp | Timescale | Outcomes | Diagnostic method/tool | Violence type | Statistics (significant in bold)<br>OR, p value, other |
|---|---|---|---|---|---|---|
| Afifi et al (2011)[25] | DV | Lifetime | Problems in movement last 4 weeks | Participant reported | Any | Few OR 1.3 (1.0-1.6)<br>**Some OR 2.8 (2.1-3.6)**<br>**Many OR 3.0 (1.7-5.2)**<br>Unable to move OR 1 (0.1-11.1)<br>**p=0.000** |
| Al-Serkal et al (2014)[33] | IPV | Lifetime | Many problems walking / unable to walk | Not documented | Physical AND sexual<br>Physical ONLY | **OR 7 (2.5-19.7)**<br>**OR 3.7 (1.2-11)** |
| Eldoseri et al (2014)[49] | IPV | Lifetime | Problems with movement in the last 4 weeks | Participant reported. WHO Violence against Women questionnaire (version 10.0) – adapted version | Physical | OR 1.4 (0.7-2.6)<br>p=0.3 |

## Medication use

| Paper | Perp | Timescale | Outcomes | Diagnostic method/tool | Violence type | Statistics (significant in bold)<br>OR, p value, other |
|---|---|---|---|---|---|---|
| Afifi et al (2011)[25] | DV | Lifetime | Took drugs in the last 4 weeks | Participant reported | Any | **OR 2.4 (2.0-2.9)**<br>**p=0.000** |
| Al-Modallal (2012)[27] | IPV | 12m | Use of prescribed tranquillizers | Participant reported | Any | **aOR 0.014 (0.00-0.86)** |
| Eldoseri et al (2014)[49] | IPV | Lifetime | Taken pain medication in the last 4 weeks | Participant reported. WHO Violence against Women questionnaire (version 10.0) – adapted version | Physical | OR 1.09 (0.6-2.1)<br>p=0.8 |

**Other general health problems with significant associations found by studies:** headache,[62] heart problems,[30] movement problems,[25] problems walking,[33] epilepsy,[42] chronic disease,[42] gastrointestinal problems,[30,41,62] respiratory problems,[30,62] urinary problems,[30,62] liver problems,[30] smoking/addiction/alcohol.[54]

**Other general health problems with no evidence of significant associations found by studies:** headache,[41] arthritis,[30] heart problems,[30] movement,[49] gastrointestinal,[30] respiratory,[30] urinary,[30] kidney problems,[30] smoking.[27]
